# CD45RA and CD45RO Are Regulated in a Cell-Type Specific Manner in Inflammation and Sepsis

**DOI:** 10.3390/cells12141873

**Published:** 2023-07-17

**Authors:** Muhammad G. T. Ahmed, Andreas Limmer, Matthias Hartmann

**Affiliations:** 1 Department of Anesthesiology and Intensive Care Medicine, University Hospital Essen, University Duisburg-Essen, 45147 Essen, Germany; muhammadthabet87@aun.edu.eg (M.G.T.A.); andreas.limmer@uk-erlangen.de (A.L.); 2 Anesthesia, Intensive Care and Pain Management, South Egypt Cancer Institute, Assiut University, Assiut 7111, Egypt

**Keywords:** CD45, CD45RA, CD45RO, inflammation, sepsis, lipopolysaccharides, granulocytes, lymphocytes, monocytes

## Abstract

CD45 is a transmembrane glycoprotein that is located on the surface of all leukocytes and modulates both innate and adaptive immune system functions. In a recent study, inflammation modulated the CD45 expression in leukocytes, but the effect on the expression of CD45 subtypes is unknown. In the present study, we therefore investigated the effect of inflammatory conditions in humans (surgery, sepsis) and ex vivo incubation with lipopolysaccharides (LPS) on the expression of the subtypes CD45RA and CD45RO in granulocytes, lymphocytes, and monocytes. Whole blood samples were obtained from healthy volunteers, postoperative patients, and patients with sepsis at day 1 of diagnosis, respectively. Samples were incubated with fluorescent antibodies directed against CD45, CD45RA and CD45RO in the absence and presence of lipopolysaccharide and subjected to flow cytometry. In comparison to volunteers, CD45RA surface expression in postoperative and septic patients was reduced by 89% exclusively on granulocytes, but not on lymphocytes or monocytes. In contrast, CD45RO was exclusively reduced on lymphocytes, by 82%, but not on other cell types. Receiver operating characteristic curve analyses demonstrated that CD45RA (on granulocytes) and CD45RO (on lymphocytes) allow a good differentiation of volunteers and patients with sepsis (AUC = 0.9; *p* = 0.0001). The addition of LPS to the whole blood samples obtained from volunteers, postoperative patients, and septic patients markedly increased the CD45RO expression in granulocytes, lymphocytes, and monocytes. In contrast, LPS reduced CD45RA exclusively on monocytes. In conclusion, the surface expression of CD45RA and CD45RO is regulated in inflammation in a cell-type- and stimulus-specific manner. Considering that CD45 subtypes are critically involved in immune system signaling, the pathophysiologic and diagnostic implications warrant further investigation.

## 1. Introduction

CD45 is a glycoprotein with protein phosphatase activity abundantly expressed on the surface of all leukocytes [1,2,3]. The protein is highly preserved during evolution and has been demonstrated even in chicken, sharks, and mosquitos [4]. CD45 increases T-cell and B-cell receptor signaling as well as the development and function of leukocytes [5]. Moreover, CD45 can reduce Toll-like receptors and cytokine signaling, as well as cell adhesion and migration in a cell type specific manner (for a review see [6]). Mutations in CD45 can be accompanied by severe combined immune disease [7,8,9]. In humans, six splice variants differing in the extracellular part of the molecule have been demonstrated [1,10,11]. The human variants are termed CD45RABC (also named CD45R), CD45RAB, CD45RBC, CD45RA, CD45RB, and CD45RO, and are detected by subtype specific antibodies and molecular weight, respectively (for review see [1]). The term CD45 includes all subtypes and is detected by an antibody binding to all subtypes. The knowledge of differences in functions of CD45 subtypes, which are probably caused by different glycosylation and sizes of the extracellular N-terminus, is just beginning to evolve [1].

Many substrates of the protein tyrosine phosphatase have been demonstrated under in vitro conditions. In vivo, when cellular compartmentation is intact, CD45 dephosphorylates Src family kinases, Src family kinase substrates, and Janus kinases [12,13]. Interestingly, there is a lack of physiological ligands of CD45. The only available information suggests specific binding to (1) placental protein 14, (2) UL11, a glycoprotein from cytomegalovirus, and (3) E3/49K, a protein secreted by adenovirus infected cells [14,15,16,17].

Due to the lack of physiological ligands, it is thought that the amount of CD45 dictates the immunoregulatory response of the glycoprotein, too [6]. Taken together, the findings suggest that CD45 modulates immune system function (1) by the extent of its surface expression and (2) by the binding of certain ligands, respectively.

In humans, two gene polymorphisms have been detected in the PTPCR gene encoding CD45, and they are associated with several diseases. C77G is associated with immune disorders including autoimmune hepatitis, HIV infection, and multiple sclerosis, while A138G is associated with hepatitis B and Graves’ disease [1,18].

Alterations in the surface expression of CD45 are associated with the outcome of several hematological malignancies including chronic lymphatic leukemia, Hodgkin’s disease, childhood acute lymphatic leukemia, multiple myeloma, and diffuse large B cell lymphoma (for a review, see [1]). Moreover, a downregulation of CD45 signaling in peripheral blood mononuclear cells obtained from patients with COVID-19 and breast cancer, respectively, has recently been demonstrated [19,20].

Little information is available on the eventual regulation of CD45 expression. In a recent study, we demonstrated that CD45 surface expression is altered in experimental endotoxemia and in patients with COVID-19 in a leukocyte-subtype-specific way [21]. In another study, the expression of two CD45 subtypes, CD45RA and CD45RO, was differentially regulated upon stimulation with phytohemagglutinin in an experimental setting [22]. It is thus conceivable that inflammation might affect the surface expression of CD45 subtypes in a leukocyte-subtype- and CD45-subtype-specific manner.

Therefore, we investigated the effect of inflammation caused by surgery, sepsis, and lipopolysaccharides (LPS), respectively, on the expression of CD45 subtypes. In detail, we compared the surface expression of CD45, CD45RA, and CD45RO in granulocytes, lymphocytes, and monocytes in volunteers, postoperative patients, and patients with sepsis. Moreover, we spiked the samples with LPS (ex vivo) to investigate the short-term effects of TLR4 stimulation on the surface expression of CD45, CD45RA, and CD45RO.

## 2. Materials and Methods

After approval by the local ethics committee (17-7824-BO and additional amendment), blood was drawn from volunteers, postoperative patients, and patients with sepsis, diagnosed using the sepsis-3 criteria [23].

Then, 2.7 mL lithium heparin whole blood samples were drawn and aliquots with final volumes of 50 µL were incubated with LPS 50 ng/mL from Escherichia coli (O111:B4, Sigma-Aldrich, St. Louis, MO, USA) and vehicle, respectively (60 min at 37 °C). Samples were incubated with antibodies directed against CD45, CD45RA, and CD45RO, as well as CD14, for 15 min at 23 °C. In detail, PerCP/Cyanine5.5 anti-human CD45, isotype mouse IgG1 (1 µg/mL final concentration), APC anti-human CD45RA, isotype Mouse IgG2b (2 µg/mL final concentration), PE anti-human CD45RO, isotype Mouse IgG2a (2 µg/mL final concentration), and PE/Cyanine7 anti-human CD14 isotype Mouse IgG1 (2 µg/mL final concentration) were obtained from Biolegend, San Diego, CA, USA. Thereafter, erythrocytes were lysed with 0.5 mL RBC lysis buffer (from Pluriselect, Leipzig, Germay) for 10 min at 4 °C.

**Figure 1:**
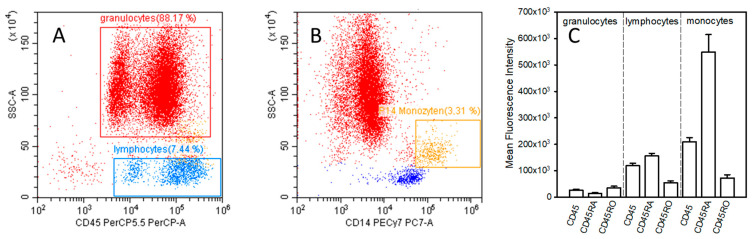
Diagrams (**A**,**B**) demonstrate the gating of granulocytes, lymphocytes, and monocytes. Diagram (**C**) shows the surface expression of CD45, CD45RA, and CD45RO in healthy volunteers. For the gating sideward scatter area (SSC-A), CD45 and CD14 were used.

After the lysis of erythrocytes, samples were subjected to flow cytometric analysis (CytoFlex Flow Cytometer, Beckman Coulter, Inc., Brea, CA, USA). Routine daily quality control of analysis was carried out using cytoflex fluorospheres from Beckmann Coulter to assure the sustained validity of measurements. For the detection of leucocyte subtypes, gates were defined by the use of side-scatter as well as CD45 and CD14 expression (PerCP/Cyanine5.5 fluorescence intensity). Gating of granulocytes, lymphocytes, and monocytes is shown in Figure 1A,B. For the evaluation of the surface expression of CD45, CD45RA, and CD45RO, the mean fluorescence intensity (MFI) in granulocytes, lymphocytes, and monocytes was determined. Analysis was carried out with the device’s software (CytExpert version 2.4.0.28, Beckman Coulter, Inc., Brea, CA, USA).

For the statistical evaluation and generation of graphs, SPSS (Version 23, IBM, Armonk, NY, USA) and Prism (version 8.4.3, GraphPad software, Boston, MA, USA) were used. ANOVA was used to evaluate the eventual significance of differences in volunteers, postoperative patients, and patients with sepsis. When the ANOVA showed significant differences between groups (*p* < 0.05), the Student’s *t*-test combined with Bonferroni correction was used as the post hoc test. Data are given as mean and standard error of the mean. Moreover, receiver operating characteristic (ROC-) curves, area under the curve, and asymptotic significance levels were used to evaluate the ability of CD45 subtypes to discriminate between volunteers and patients with sepsis.

## 3. Results

### 3.1. Patients’ Characteristics

CD45, CD45RA, and CD45RO were measured in granulocytes, lymphocytes, and monocytes obtained from 20 patients with sepsis; the surface expression in volunteers is shown in Figure 1C. Causes for sepsis included pneumonia as the leading cause in thirteen patients, followed by urosepsis in four cases. The cause was unknown in the remaining three cases. Sepsis was diagnosed according to Singer et al., 2016 [23]. Postoperative patients investigated in the present study had undergone major surgery with laparotomy. As the control group, healthy volunteers were investigated.

### 3.2. Expression of CD45, CD45RA, and CD45RO in Granulocytes

**Figure 2:**
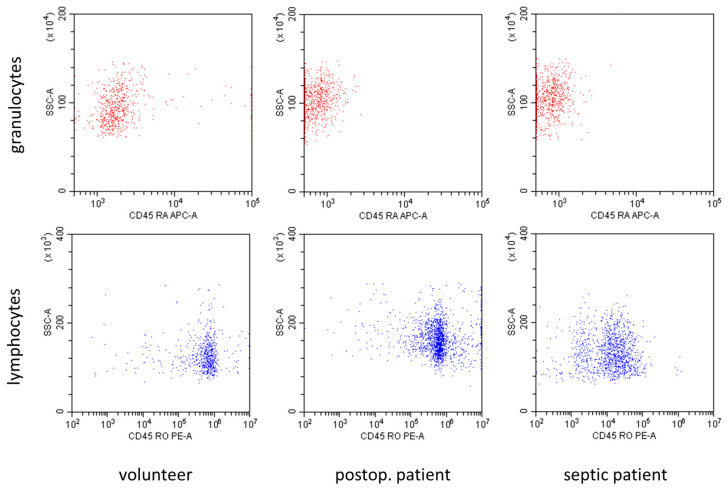
Representative flow cytometry diagrams demonstrating the expression of CD45RA in granulocytes and the expression of CD45RO in lymphocytes in volunteers, postoperative patients, and patients with sepsis. Expression of both subtypes was markedly reduced in patients with sepsis.

In Figure 2, representative flow cytometry diagrams showed marked differences in the expression of CD45 subtypes in volunteers and patients with sepsis. The CD45RA expression in granulocytes, as well as the CD45RO expression in lymphocytes, decreased in patients with sepsis in comparison to volunteers.

**Figure 3:**
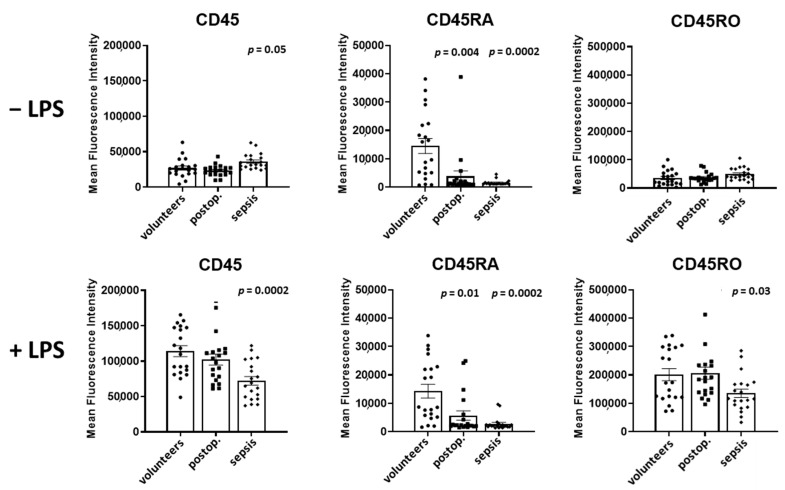
Granulocyte CD45, CD45RA, and CD45RO expression in volunteers, postoperative patients, and patients with sepsis in absence of LPS (**upper** lane) and presence of LPS (**lower** lane). The mean fluorescence intensity was determined using flow cytometry of antibody treated cells. Results are shown as individual data points as well as mean and standard error of the mean. *p*-values denote Bonferroni corrected *t*-test results as the post hoc test performed subsequent to a significant ANOVA result.

In Figure 3, the surface expression of CD45, CD45RA, and CD45RO, presented as mean fluorescence intensity, on granulocytes in volunteers, postoperative patients, and patients with sepsis in both unstimulated and LPS-stimulated samples obtained from 20 persons per group is shown.

In the absence of LPS, the expression of CD45 in granulocytes was unaltered in postoperative patients but increased by 33% in patients with sepsis in comparison to volunteers (*p* = 0.05).

Spiking samples with LPS led to a 4.2-fold increase in CD45 in volunteers and to a 4.4-fold increase in postoperative patients (*p* = 0.0002 for both groups). In patients with sepsis, LPS induced an only 2.0-fold increase in CD45 (*p* = 0.0002).

The expression of CD45RA was reduced by 74% in postoperative patients (*p* = 0.004) and by 89% in septic patients when compared to volunteers (*p* = 0.0002). The spiking of the samples with LPS increased the CD45RA expression exclusively in patients with sepsis by 79% (*p* = 0.004).

The CD45RO expression of granulocytes was not different in volunteers, postoperative and septic patients. The spiking the samples with LPS increased the expression of CD45RO in volunteers (5.7-fold, *p* = 0.0002), postoperative patients (5.6-fold, *p* = 0.0001), and patients with sepsis (2.7-fold, *p* = 0.0002).

A comparison of CD45 subtypes in LPS-incubated samples demonstrated a decreased expression of CD45, by 37% (*p* = 0.0002), of CD45RA, by 81% (*p* = 0.0002), and of CD45RO, by 33% (*p* = 0.03), in patients with sepsis in comparison to volunteers.

### 3.3. Expression of CD45, CD45RA, and CD45RO in Lymphocytes

**Figure 4:**
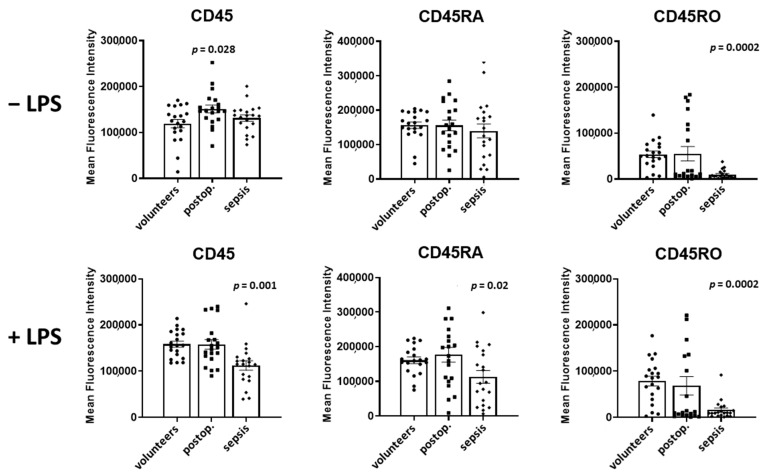
Lymphocyte CD45, CD45RA, and CD45RO in volunteers, postoperative patients, and patients with sepsis in absence of LPS (**upper** lane) and presence of LPS (**lower** lane) Cells were labeled with antibodies and fluorescence intensity was measured using flow cytometry. Results are shown as individual data points as well as mean fluorescence intensity and standard error of the mean. *p*-values denote Bonferroni corrected *t*-test results as the post hoc test performed subsequent to a significant ANOVA result.

In lymphocytes, CD45 levels in volunteers and patients with sepsis were not different, but increased by 27% in postoperative patients (*p* = 0.03) (Figure 4). The spiking of samples with LPS increased the CD45 expression in volunteers by 33% (*p* = 0.0004), had no significant effect in postoperative patients, and decreased the CD45 expression in patients with sepsis by 14% (*p* = 0.001).

The CD45RA levels were not different in the three groups in the absence of LPS. The spiking of the samples with LPS did not affect the CD45RA expression in volunteers and postoperative patients, but reduced the CD45RA expression by 19% in patients with sepsis (*p* = 0.012).

In comparison to volunteers, CD45RO was markedly reduced by 82% in septic patients (*p* = 0.0002). LPS increased the CD45RO expression in volunteers by 46% (*p* = 0.0002), in postoperative patients by 24% (*p* = 0.03), and in patients with sepsis by 61% (*p* = 0.04).

A comparison of CD45 subtypes in LPS-incubated samples of volunteers and patients with sepsis demonstrated decreased expressions (in sepsis) of CD45 by 29% (*p* = 0.001), CD45RA by 30% (*p* = 0.02), and CD45RO by 80% (*p* = 0.0002).

### 3.4. Expression of CD45 Subtypes in Monocytes

**Figure 5:**
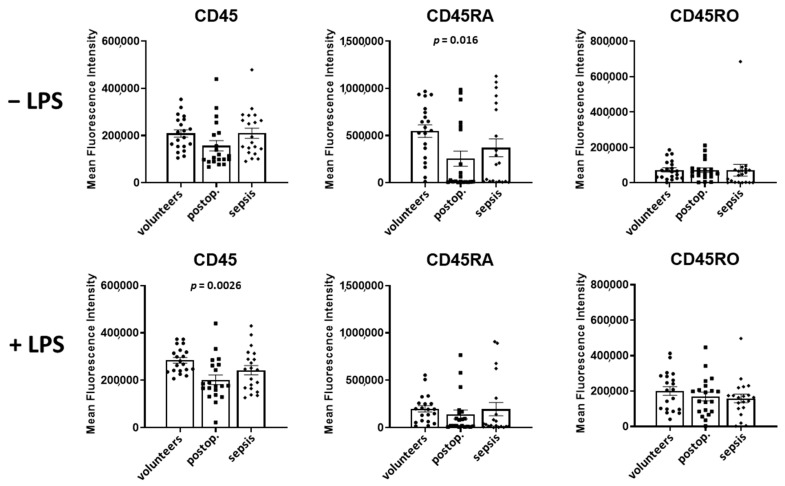
Monocyte CD45, CD45RA, and CD45RO in volunteers, postoperative patients, and patients with sepsis in absence of LPS (**upper** lane) and presence of LPS (**lower** lane). Whole blood samples were incubated with antibodies and subjected to flow cytometry. Data of the mean fluorescence intensity are shown as individual data points as well as mean and standard error of the mean. *p*-values denote Bonferroni corrected *t*-test results as the post hoc test performed subsequent to a significant ANOVA result.

As shown in Figure 5, CD45 expression on the surface of monocytes was not different in volunteers, postoperative and septic patients in the absence of LPS. However, LPS increased the expression of CD45 in volunteers by 36% (*p* = 0.002), and in postoperative patients by 29% (*p* = 0.04).

In comparison to the volunteers, CD45RA expression was not different in patients with sepsis, but decreased in postoperative patients by 53% (*p* = 0.016). Spiking the samples with LPS led to a marked decrease in CD45RA expression in volunteers, by 64% (*p* = 0.0002), by 46% in postoperative patients (*p* < 0.04), and by 48% in septic patients (*p* = 0.012).

CD45RO expression was not different in volunteers, postoperative patients, and patients with sepsis. In the presence of LPS, CD45RO expression increased 2.8-fold in volunteers (*p* = 0.0002), 2.4-fold in postoperative patients (*p* = 0.0002), and 2.2-fold in postoperative patients (*p* = 0.0008).

### 3.5. Receiver Operating Characteristic Curve Analyses

**Figure 6:**
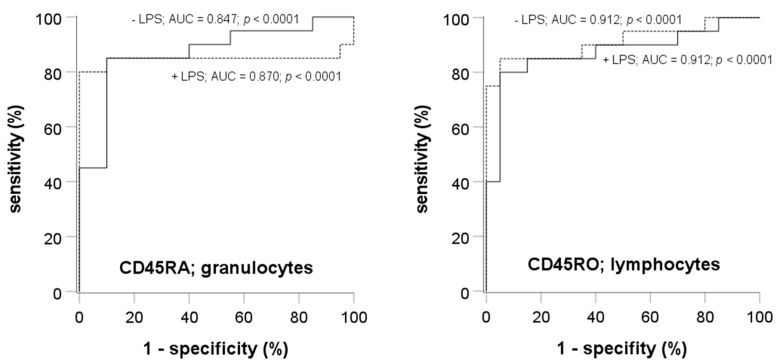
Receiver operating characteristic curves, area under the curve, and asymptomatic significance levels demonstrating the capability of CD45RA in granulocytes and CD45RO in lymphocytes to differentiate between volunteers and patients with sepsis. Addition of LPS did not affect the excellent discrimination between volunteers and patients with sepsis.

The marked changes in CD45RA in granulocytes and CD45RO in lymphocytes suggested that these surface markers might serve as biomarkers for the diagnosis of sepsis. To further evaluate the diagnostic value, we calculated the receiver operating characteristic curves. The results, shown in Figure 6, reveal a good discrimination with both CD45RA and CD45RO in both the absence and presence of LPS. The area under curve was in the range between 0.847 and 0.912, and the asymptotic significance level was always *p* < 0.0001.

### 3.6. Distribution of Leucocyte Subtypes in Volunteers, Postoperative Patients, and Patients with Sepsis

**Figure 7:**
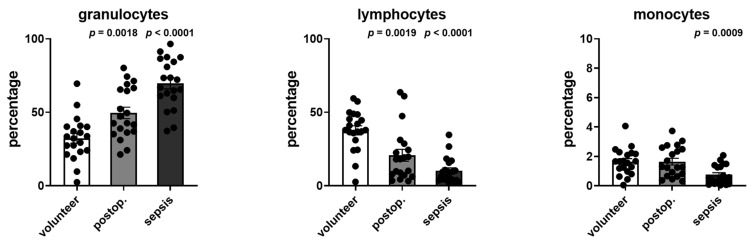
Distribution of granulocytes, lymphocytes, and monocytes in volunteers, postoperative patients, and patients with sepsis. Data are given as the percentage of the respective leucocyte subtype to all subtypes (100%) as gated with flow cytometry. *p*-values denote Bonferroni corrected *t*-test results as the post hoc test performed subsequent to a significant ANOVA result.

The distribution of leukocytes, lymphocytes, and monocytes was determined from the cell cytometry data. The changes in the distribution are shown in Figure 7 and demonstrated an increase in the percentage of granulocytes as well as decreases in lymphocytes and monocytes.

## 4. Discussion

The present study demonstrates that CD45RA and CD45RO are regulated in inflammation in a cell-specific manner, as evidenced in volunteers, surgical patients, and patients with sepsis, as well as in ex vivo LPS-treated samples. In granulocytes, CD45RA (but not CD45RO) decreased in postoperative patients and patients with sepsis. In contrast, in lymphocytes, CD45RO (but not CD45RA) decreased in sepsis. In monocytes, neither CD45RA nor CD45RO were altered in sepsis. Ex vivo, LPS-incubation markedly increased the CD45RO expression of granulocytes, lymphocytes, and monocytes in volunteers, postoperative patients, and septic patients. CD45RA was decreased in LPS-treated samples, exclusively in monocytes. The measurement of CD45RA in granulocytes and CD45RO in lymphocytes allowed the differentiation of volunteers and patients with sepsis with high accuracy, as evidenced by ROC-analyses.

The observed alterations in the expression of CD45 subtypes are an important finding, as the constitutively active protein tyrosine phosphatase regulates the phosphorylation state of certain phosphoproteins, including Src family kinases, Src family kinase substrates, and Janus kinases [6]. The exact differences in function of CD45 subtypes are largely unknown, but variances in signal transduction due to the size and extent of glycosylation of the molecule’s extracellular part have been demonstrated [11,24].

Many experimental studies indicate that CD45 is an immunoregulatory glycoprotein affecting the immune response in T-cells, B cells, and macrophages [5,6]. Interestingly, the amount of CD45 molecules, and not eventual receptor ligands, are thought to modulate the dephosphorylation of phosphoproteins and thus modulate cellular responses [6]. This assumption is based on the fact that only few ligands of CD45 have been detected: a placental protein (placental protein 14), a viral protein from cytomegalovirus (UL11), and E3/49K secreted from adenovirus infected cells have been described [14,15,16,17]. In addition to those specific ligands, lectins have been demonstrated to bind rather unspecifically to CD45 and to modulate its function [1].

Concerning the function of CD45 in humans, there is increasing evidence indicating important physiological functions in innate and adaptive immune system function [1]. Two human gene polymorphisms of CD45 are associated with several diseases with immune system involvement [1]. Moreover, mutations of CD45 are associated with severe immune defects, the occurrence of viral infections, and autoimmune diseases [9]. In a recent pilot study, we demonstrated that CD45 is regulated in COVID-19 patients [21]. However, in that study the involvement of the CD45 subtypes was not investigated. In the present study, we investigated the effect of inflammation on CD45RA and CD45RO, which have been shown to be affected by phytohemagglutinin in T-cells in vitro [22]. The present study extends knowledge on the regulation of CD45, demonstrating that there is a complex regulation of CD45RA and CD45RO expression. The regulation in inflammation shows marked differences between CD45RA and CD45RO, as well as the investigated leukocyte subtypes. Moreover, either up- or downregulation was shown to be dependent on the inflammatory stimulus. It can be hypothesized that the complex regulation of CD45RA and CD45RO surface expression reflects specific physiological functions of the CD45 subtypes varying between leucocyte subtypes. However, there is currently no information available explaining the physiological importance of the observed regulation of the CD45 subtypes. However, an immunomodulatory effect is probable.

There have been many attempts made to use flow cytometry for the diagnosis of sepsis (for a review, see [25]). An association of several surface molecules with immune suppression has been demonstrated so far, including mHLA-DR, immature neutrophils and/or MDSC count, lymphocyte count, and regulatory lymphocytes, as well as PD-1 expression. Moreover, many studies have demonstrated a complex regulation of the leucocyte count in sepsis (for a review, see [26]). The present studies only differentiate between granulocytes, lymphocytes, and monocytes, but our results are in line with those studies. The first multicentric studies using flow cytometry have been initiated. In view of the above stated progress in the clinical use of flow cytometry in translational sepsis research, the present study demonstrates that CD45 subtypes might serve as biomarkers for immune modulation in inflammation and sepsis in clinical studies.

The present study has limitations. Although the results are highly significant, the number of patients is limited and investigations in a greater collective seem necessary. Moreover, an eventual association with outcome variables has to be demonstrated. In addition, a more detailed evaluation of leukocyte subtypes seems desirable. Likewise, the investigation of the time course of changes in the expression of subtypes, the involved signal transduction pathways, and the functional consequences on the cell level warrant further investigations.

## 5. Conclusions

The present study adds important knowledge to the physiology of CD45 subtypes in humans, demonstrating leucocyte-specific alterations in the expression of the protein tyrosine phosphatase subtypes in inflammation and sepsis. While the findings of the present study demonstrate that CD45 subtypes might be suitable biomarkers in inflammation and sepsis, the functional importance of this has to be demonstrated in further studies.

## Data Availability

Data can be provided on reasonable request.
